# Changes in the shape and function of the fetal heart of pre- and gestational diabetes mothers

**DOI:** 10.1186/s12884-024-06262-z

**Published:** 2024-01-11

**Authors:** Shuang Li, Linlin Wang, Huixia Yang, Lixin Fan

**Affiliations:** Department of Obstetrics and Gynecology, Peking University First Hospital, Beijing, China

**Keywords:** Hyperglycemia in pregnancy, Fetal echocardiography, Cardiac function, Fetal HQ, Maternal metabolic indices

## Abstract

**Background:**

Hyperglycemia during pregnancy can affect fetal heart in many ways, including causing cardiac malformation, leading to hypertrophic cardiomyopathy and cardiac dysfunction. Echocardiographic evaluation can assist identify alterations in heart structure, morphology and function, enabling prompt monitoring and management. However, according to earlier research, the cardiac alterations are modest in hyperglycemic mothers’ fetuses, and might not be detectable using conventional methods and it is also unclear whether these changes are related to the metabolism of mothers. Fetal Heart Quantification (Fetal HQ) can assess ventricular geometry and function more sensitively and thoroughly, and identify sub-clinical cardiac dysfunction. The purpose of this study was to evaluate fetal heart by Fetal HQ in fetuses of hyperglycemic mothers who either had pre-gestational or gestational diabetes and to correlate them with maternal metabolic indices.

**Methods:**

The fetuses of 25 gestational age-matched control mothers, 48 women with gestational diabetes mellitus (GDM), and 11 women with diabetes mellitus (DM) were included in the prospective case-control research. Using fetal echocardiography and speckle tracking echocardiography (STE), the heart of the fetus was evaluated. Differences in the groups’ anthropometric, metabolic, and cardiac parameters were examined. It was assessed whether maternal features, prenatal glucose, lipids, and maternal hemoglobin A1c (HbA1c) correlated with fetal cardiac parameters.

**Results:**

The LV EDV and ESV were significantly higher in the GDM group as compared to the DM group (*p* < 0.05). The GSI% was significantly lower in the GDM group compared with the control (*p* < 0.05). The LV SV and CO of the GDM group were both significantly higher compared with the DM group (*p* < 0.05). There was a significant decrease in RV FS for segments 1–7 in GDM fetuses compared to the control (*p* < 0.05) and for segments 5–10 compared to DM (*p* < 0.05). Fetal cardiac morphology and function indices correlate with maternal pregestational weight, BMI, early pregnancy fast glucose, lipids, and glycemic control levels.

**Conclusions:**

Fetuses exposed to gestational diabetes have altered heart morphology and function that is linked to maternal metabolic parameters, which presents a special indication for performing geometry and function cardiac assessment. Fetal HQ can be employed to evaluate the fetal cardiac shape and function in fetuses exposed to gestational diabetes.

## Background

Hyperglycemia during pregnancy is turning into a significant health issue in the modern day due to the rise in the number of women reaching childbearing age and the global obesity pandemic. According to some studies, maternal hyperglycemia can affect the morphology of the heart, which can result in hypertrophic cardiomyopathy [1, 2], as well as cardiac dysfunction even in the absence of structural abnormalities [2–4] and cardiac hypertrophy [5, 6]. All of these impacts could have a considerable perinatal morbidity and death rate as well as long-term effects on children’s cardiovascular systems [7–9].

It is unknown how maternal hyperglycemia affects the architecture and function of the fetal heart, and it is also unclear whether these cardiac abnormalities are related to the metabolism of the mother. Analysis of fetal heart function might offer crucial information about fetal hemodynamics and cardiovascular adaptation due to the influence of numerous perinatal problems, such as hyperglycemia in pregnancy.

Over the past few years, fetal echocardiography has developed into a crucial non-invasive technique for assessing fetal cardiac anatomy, hemodynamics, and function. It is a reliable method for detecting cardiac malformations and measuring fetal cardiac function [10–12]. However, the conventional echocardiographic evaluation methods provide only indirect information on systolic and diastolic function and myocardial deformation is not directly measured. New approaches from adult cardiology can be used for fetuses as fetal cardiac imaging develops. Such as Fetal Heart Quantification (Fetal HQ), a new two-dimensional speckle tracking technology that allows a direct or offline measurement to quantitatively evaluate the overall and segmental deformity and cardiac function of fetuses. This technology is relatively angle-independent and enables a more sensitive and thorough assessment of ventricular geometry and function, which aids in the identification of sub-clinical abnormalities of diastolic and systolic function [11, 13–15].

The purpose of this study was to compare the cardiac geometry and function of fetuses from diabetic mothers to those from controls utilizing Fetal HQ, as well as to link the results with maternal glucose and lipid metabolism parameters.

## Methods

### Study population

In this prospective observational study, which took place between December 2020 and March 2022 at Peking University First Hospital, a tertiary care facility in Beijing, China, pregnant women who were receiving prenatal routine care during their second trimester were recruited as subjects.

The study comprised pregnant women with DM or GDM according to Chinese guidelines for the diagnosis and treatment of hyperglycemia in pregnancy [16], pregestational and gestational diabetes were diagnosed with a single 75 g glucose tolerance test. The top limits for blood sugar during fasting and blood sugar one and two hours after consuming glucose were 5.1, 10.0, and 8.5 mmol/l, respectively. After a normal glucose tolerance test, non-diabetic pregnant women whose fetal hearts are anatomically and functionally normal were added to the control group.

Exclusion criteria for the study included pregnant women who had pre-eclampsia, intrauterine growth restriction, fetal congenital deformities, known genetic and chromosomal disorders, and abnormal fetal heart rhythms (either tachycardia or bradycardia) at the time of enrollment.

### Echocardiographic measurements

A fetal echocardiogram was performed by two skilled professionals (L.W. and H.Z.) to assess the structure and function of fetal hearts. The latest version of the General Electric Voluson E10 BT20 ultrasound system in our department was used to perform speckle tracking analysis utilizing the Fetal HQ software by H.Z. who has received formal training of performing and interpreting the procedures. Based on the methodology previously described [13, 15, 17–20], the global sphericity index (GSI), global longitudinal strain (GLS), fractional area change (FAC) for both ventricles, left ventricular (LV) ejection fraction, LV stroke volume, LV cardiac output, 24-segment end-diastolic diameter (EDD), 24-segment transverse fractional shortening (FS), and 24-segment sphericity index (SI) for both ventricles in absolute values and Z-score were measured and calculated.

### Statistical analyses

The normality test (Kolmogorov–Smirnov) was performed on the quantitative variables. The normal distribution variables were presented as mean ± standard deviation and non-normal distribution variables were shown as median (interquartile range). The Kruskal-Wallis test was used to examine the non-parametric differences between variables in the three groups with non-normally distributed data and pairwise comparisons between individual groups were made when differences were found. T-pair tests with Bonferroni multiple comparison adjustments were used to analyze differences for normally distributed variables. The Spearman correlation coefficient was used to assess the relationships between maternal traits and metabolic parameters with fetal cardiac indices, and *P* < 0.05 was considered significant. Statistical analysis was performed using commercially available software, SPSS, version 26.0.

## Results

### Characteristics of the participant

The study recruited 11 DM, 48 GDM and 25 control subjects who underwent echocardiography between 20 and 38 weeks of gestation and there was no significant difference in gestational age at scans among the three groups. The mean maternal age, parity, and parity were also similar in the three groups. The pregestational weight and BMI of the mothers were statistically higher in the GDM group (*p* < 0.05) (Table 1).

**Table 1:** Demographic characteristics and pregnancy outcomes

Variable	DM (*N* = 11)	GDM (*N* = 48)	Control (*N* = 25)	P
Age	33.91 ± 4.16	34.52 ± 4.31	33.12 ± 4.23	0.424
Pre-gestational weight (Kg)	62.40(53.00–68.00)	58.35(52.73–67.43)^*^	53.00(50.75–59.6)	**0.015**
Stature (cm)	164.32 ± 4.42	162.29 ± 4.47	161.02 ± 4.17	0.117
Pre-gestational BMI (Kg/cm^2^)	22.77(20.69–25.28)	23.26(19.97–25.92)^*^	20.70(19.53–22.73)	**0.024**
Weight at delivery (Kg)	73.50(62.70–77.00)	70.55(65.38–77.55)	68.00(65.00-73.85)	0.398
Gravidity (time)	2(1–2)	2(1–3)	2(1–2)	0.659
Parity (time)	0(0–1)	0(0–1)	0(0–1)	0.676
Triglycerides (mmol/L)	1.21(0.73–1.83)	0.88(0.70–1.26)	0.80(0.65–1.17)	0.227
Total Cholesterol (mmol/L)	4.13 ± 1.16	3.96 ± 0.69	4.12 ± 0.74	0.636
HDL-Cholesterol (mmol/L)	1.38 ± 0.33	1.41 ± 0.25	1.48 ± 0.29	0.431
LDL-Cholesterol (mmol/L)	2.54 ± 0.95	2.07 ± 0.57	2.12 ± 0.44	0.065
Early pregnancy fast glucose (mmol/L)	5.69(4.96–6.58)**^**	5.07(4.94–5.46)^*^	4.87(4.62–5.14)	**0.001**
Gestational age at delivery (weeks)	38.86(38.14–39.29)^^^	39.29(38.64–39.86)	40.14(39.00–41.00)	**0.007**
Gestational age at scans (weeks)	24.43(23.86–27.57)	26.86(26.00-30.32)	25.43(22.86–29.93)	0.100
HbA1c (%)	5.81 ± 0.18	5.21 ± 0.05		**0.000**
C-section	5(45.5%)	24(50.0%)	11(44.0%)	0.878
Birth weight (Kg)	3320(3060–3760)	3432.5(3026.3-3717.5)	3190 (3045-3527.5)	0.480
APGAR at 1 min	10(10–10)	10(10–10)	10(10–10)	0.614
APGAR at 5 min	10(10–10)	10(10–10)	10(10–10)	0.468

N: the number of fetal echocardiography results, HbA1c data were not available on one GDM women

DM, diabetes mellitus; BMI, body mass index; HbA1c, hemoglobin A1c; HDL-Cholesterol, high density lipoprotein cholesterol.; LDL-Cholesterol, low-density lipoprotein cholesterol

Data presented as number (%) or median (interquartile range) or mean ± SD

^*^p < 0.05 GDM vs. control; ^#^*p* < 0.05 GDM vs. DM; ^^^*p* < 0.05 DM vs. control

Only statistically significant results after correction for multiple comparisons if *p* < 0.0167 are provided in parenthesis

Bold p values are significant

In the DM group, 1(9.1%) pregnant woman was controlled on diet alone, 8(72.7%) were on insulin and 2(18.2%) were on both oral medications and insulin. In the GDM group, 40(83.3%) were controlled on diet alone, and 8(16.7%) were on insulin.

### Metabolic parameters and pregnancy outcomes

The early pregnancy fast glucose was statistically higher in the DM group (5.69 vs. 4.87 mmol/L, *p* = 0.009) and the GDM group (5.07 vs. 4.87 mmol/L, *p* = 0.002). There were no significant differences in early pregnancy lipids in the three groups (Table 1). The mean HbA1c of the DM group was 5.81 ± 0.18% and was 5.21 ± 0.05% of the GDM group. HbA1c data were not available for one GDM woman (Table 1).

The gestational weeks for delivery were statistically earlier in the DM group (*p* = 0.010). There were no significant differences in birth weight (EFW) (*p* = 0.480) and the APGAR scores of newborns at the 1st and 5th minutes between the groups (*p* = 0.614 and 0.468) (Table 1).

### Fetal ultrasonography findings

#### Fetal cardiac morphometry

The LV ED area was significantly higher in the GDM group compared to the DM group (1.82 vs. 1.07, *p* = 0.029). The LV ED length was significantly higher in the GDM group than in the DM (2.02 vs. 1.72, *p* = 0.035) and the control group (2.02 vs. 1.79, *p* = 0.028). The LV ES area was significantly higher in the GDM group compared to the DM (1.22 vs. 0.87, *p* = 0.036) and the control group (1.22 vs. 0.95, *p* = 0.019). The LV ES length was significantly higher in the GDM group than in the DM (1.72 vs. 1.47, *p* = 0.037) and control (1.72 vs. 1.51, *p* = 0.019). The LV EDV was significantly bigger in the GDM group compared with the DM group (1.39 vs. 0.72, *p* = 0.018). The LV ESV was significantly bigger in the GDM group than in the DM (0.71 vs. 0.53, *p* = 0.047) and the control group (0.71 vs.0.50, *p* = 0.041). As for the RV ED area and length, there were no significant differences between the three groups. The RV ES area was significantly bigger in the GDM group than in the DM group (1.28 vs.0.91, *p* = 0.039). The RV ES length was significantly higher in the GDM group than in the control group (1.62 vs.1.43, *p* = 0.042). The GDM group showed a lower GSI value than the control group, but the difference was not statistically significant. The 4CV GSI% was significantly lower in the GDM group as compared to the control (61.15 vs.81.94, *p* = 0.047), while it was not significantly different between the DM group and the control group or the GDM group. The 4CV Width ED of the GDM group was significantly higher as compared with the DM (27.76 vs.24.32, *p* = 0.011) and the control (27.76 vs.24.77, *p* = 0.016). The 4CV Length ED of the GDM group was significantly higher than the DM group (35.42 vs. 30.6, *p* = 0.032). The 4CV Area of fetuses of the GDM group was significantly higher as compared to the DM (770.53 vs.574.16, *p* = 0.022) (Table 2).

**Table 2:** Comparison of fetal cardiac morphometry findings of the patients

Variable	DM (*N* = 11)	GDM (*N* = 48)	Control (*N* = 25)	P
LV ED Area (cm^2^)	1.07(1.03–1.79)	1.82(1.51–2.01)^#^	1.56(1.07–2.02)	**0.012**
LV ED Length (cm)	1.72 ± 0.30	2.02 ± 0.35^*#^	1.79 ± 0.33	**0.006**
LV ES Area (cm^2^)	0.87(0.69–1.16)	1.22(0.99–1.39)^*#^	0.95(0.71–1.21)	**0.004**
LV ES Length (cm)	1.47 ± 0.27	1.72 ± 0.32^*#^	1.51 ± 0.29	**0.005**
LV EDV (ml)	0.72(0.56–1.26)	1.39(1.15–1.63)^#^	1.15(0.65–1.79)	**0.009**
LV ESV (ml)	0.53(0.30–0.69)	0.71(0.58–0.93)^*#^	0.50(0.32–0.80)	**0.009**
RV ED Area (cm^2^)	1.16(1.05–1.57)	1.80(1.43–2.18)	1.45(0.98–2.22)	0.036
RV ED Length (cm)	1.44(1.36–1.90)	1.89(1.75–2.11)	1.59(1.42–2.07)	0.029
RV ES Area (cm^2^)	0.91(0.66–1.04)	1.28(1.04–1.61)^#^	1.00(0.69–1.55)	**0.010**
RV ES Length (cm)	1.38 ± 0.28	1.62 ± 0.30^*^	1.43 ± 0.31	**0.010**
4CV GSI	1.26 ± 0.16	1.25 ± 0.11	1.31 ± 0.10	0.064
4CV GSI% (%)	40.46(16.46–93.85)	61.15(25.43–79.04)^*^	81.94(60.92–90.87)	**0.049**
4CV GSI ZS	1.23(0.77–1.75)	0.76(0.49–1.38)	0.91(0.49–1.33)	0.224
4CV Width ED (cm)	24.32(21.42–26.24)	27.76(25.52–31.35)^*#^	24.77(21.81–28.93)	**0.002**
4CV Length ED (cm)	30.60 ± 5.44	35.42 ± 5.41^#^	32.83 ± 5.73	**0.018**
4CV Area (mm^2^)	574.16(428.58-755.34)	770.53(608.92–908.40)^#^	619.38(488.02-845.76)	**0.007**
CTAR	0.27 ± 0.04	0.29 ± 0.05	0.27 ± 0.04	0.154

LV, left ventricle; RV, right ventricle; GSI, global spherical index; ED, end-diastole; ES, end-systole; 4CV, 4 chamber view. CTAR: cardio-thoracic area ratio

^*^Statistically significant if *P* < 0.05 are provided in parenthesis

N: the number of fetal echocardiography results

Data are given as median (interquartile range) or mean ± SD

^*^p < 0.05 GDM vs. control; ^#^*p* < 0.05 GDM vs. DM; ^^^*p* < 0.05 DM vs. control

Only statistically significant results after correction for multiple comparisons if *p* < 0.0167 are provided in parenthesis

Bold p values are significant

#### Fetal cardiac function

The GLS and FAC of the RV tend to lower in fetuses of mothers with DM and GDM as compared to the control group, but the differences were not statistically significant. The LV SV of the GDM group was significantly higher than the DM group (0.67 vs.0.37, *p* = 0.016). The LV CO of the GDM group was significantly higher as compared with the DM group (93.51 vs.53.65, *p* = 0.015) (Table 3).

**Table 3:** Comparison of fetal cardiac function findings of the patients

Variable	DM (*N* = 11)	GDM (*N* = 48)	Control (*N* = 25)	P
LV Global Strain(%)	16.03(14.31–18.50)	17.06(14.35–18.87)	15.92(14.92–20.35)	0.953
RV Global Strain(%)	15.61(14.77–17.82)	15.18(13.45–16.97)	16.29(14.74–17.61)	0.382
LV Frac. Area change(%)	31.86(28.70-35.11)	33.32(29.24–36.35)	35.38(32.25–38.81)	0.139
RV Frac. Area change(%)	32.79(25.41–34.40)	27.33(23.80-29.51)	29.60(26.19–33.19)	0.052
LV EF(%)	44.49(43.19–48.85)	45.77(42.00-52.03)	50.19(45.10-54.97)	0.071
LV SV(ml)	0.37(0.25–0.57)	0.67(0.48–0.94)^#^	0.62(0.33–0.80)	**0.017**
LV SV/Kg(ml/kg)	0.54 ± 0.06	0.61 ± 0.03	0.65 ± 0.04	0.403
LV CO(ml/min)	53.65(34.25–85.12)	93.51(64.24–125.00)^#^	87.04(45.01-113.35)	**0.015**
LV CO/KG(ml/min/kg)	78.35 ± 8.89	86.39 ± 4.21	90.29 ± 4.97	0.606

LV, left ventricle; RV, right ventricle; BMI, Body mass index; GLS, global longitudinal strain; FAC, fractional area change; EF, ejection fraction; SV, stroke volume; CO, cardiac output

Statistically significant if *P* < 0.05 are provided in parenthesis

N: the number of fetal echocardiography results. Data are given as median (interquartile range) or mean ± SD

^#^*p* < 0.05 GDM vs. DM

Only statistically significant results after correction for multiple comparisons if *p* < 0.0167 are provided in parenthesis

Bold p values are significant

#### 24-Segment analysis

The LV ED for segments 1–6 and 9–12 were significantly increased in fetuses exposed to GDM compared to DM (*p* < 0.05). The RV ED for segments 13–14 was significantly increased in fetuses exposed to GDM compared to DM (*p* < 0.05). There were no significant differences among the three groups for both ventricular 24-segment SI value or Z-Scores. There were no significant differences in the LV FS value among the three groups, while the LV FS Z-Scores for segments 2–5 in fetuses exposed to DM were significantly changed compared to the control (*p* < 0.05). The RV FS in fetuses exposed to GDM was significantly lower for segments 1–7 compared to the control (*p* < 0.05) and for segments 5–10 compared to DM (*p* < 0.05). The RV FS Z-Scores in fetuses exposed to GDM significantly changed for segments 1–8 compared to the control (*p* < 0.05) and for segments 7–11 compared to DM (*p* < 0.05) (Tables 4, 5 and 6).

**Table 4:** Comparison of ventricular 24-segment ED values among groups

LV 24 Segment ED Values(mm)					RV 24 Segment ED Values(mm)			
Segment	DM (*N* = 11)	GDM (*N* = 48)	Control (*N* = 25)	P	DM (*N* = 11)	GDM (*N* = 48)	Control (*N* = 25)	P
1	9.35 (8.59–10.25)	11.115 (9.86–13.25)^#^	10.78 (9.28–12.17)	**0.016**	10.25 (9.02–11.42)	11.93 (9.87–13.46)	11.09 (8.97–12.77)	0.187
2	9.37 (8.64–10.25)	11.145 (9.78–12.94)^#^	10.72 (9.33–12.21)	**0.022**	10.54 (8.88–11.34)	11.845 (9.86–13.76)	11.22 (9.1-12.88)	0.183
3	9.38 (8.5-10.25)	11.245 (9.68–12.75)^#^	10.46 (9.27–12.3)	**0.024**	10.85 (8.74–11.25)	11.62 (9.83–13.99)	10.94 (9.08–12.98)	0.187
4	9.41 (8.2-10.26)	11.42 (9.51–12.67)^#^	10.54 (9.28–12.37)	**0.033**	11.01 (8.78–11.15)	11.73 (9.91–14.05)	10.85 (9.07–12.98)	0.209
5	9.45 (7.97–10.45)	11.465 (9.04–12.35)^#^	10.38 (9.06–12.36)	**0.044**	10.77 (8.87–11.44)	11.665 (9.99–13.89)	10.65 (9.05–12.84)	0.206
6	9.51 (7.84–10.62)	11.455 (9.28–12.33)^#^	10.37 (8.77–12.39)	**0.041**	10.37 (8.98–11.7)	11.67 (10.1–17.70)	10.46 (9.04–13.05)	0.178
7	9.22 (7.84–10.74)	11.34 (9.35–12.52)	10.34 (8.34–12.26)	0.036	9.98 (8.98–11.84)	11.635 (10.14–13.27)	10.5 (9.01–12.95)	0.142
8	8.83 (8-10.78)	11.135 (9.28–12.05)	10.24 (8.12–12.07)	0.032	10.09 (9.07–11.71)	11.435 (9.95–13.13)	10.18 (8.77–12.66)	0.113
9	8.67 (7.98–10.69)	10.99 (9.29,11.74)^#^	10.03 (7.91,11.86)	**0.027**	9.75 (8.9-11.52)	11.4 (9.64,13)	9.83 (8.56,12.21)	0.073
10	8.55 (7.91–10.47)	10.785 (9.3-11.57)^#^	9.94 (7.89–11.66)	**0.027**	9.4 (8.63–11.24)	11.195 (9.28–12.51)	9.61 (8.40-11.93)	0.057
11	8.56 (7.63–10.12)	10.5 (9.43–11.31)^#^	9.49 (7.91–11.47)	**0.022**	9.06 (8.27–10.79)	10.885 (9.14–12.1)	9.44 (8.1-11.27)	0.033
12	8.67 (7.3–9.69)	10.295 (9.18–11.27)^#^	9.19 (7.95–11.25)	**0.026**	8.72 (8.02–10.21)	10.51 (8.92–11.58)	9.16 (7.81–10.91)	0.017
13	8.71 (7.14–9.27)	9.93 (8.84–11.15)	9.09 (7.93–11.02)	0.037	8.38 (7.73–9.68)	10.13 (8.68–11.13)^#^	8.83 (7.47–10.45)	**0.014**
14	8.63 (6.98–8.87)	9.58 (8.37–10.99)	8.86 (7.85–10.72)	0.042	8.05 (7.37–9.29)	9.72 (8.43–10.72)^#^	8.46 (7.11–9.92)	**0.012**
15	8.2 (6.8–8.71)	9.235 (8.17–10.73)	8.57 (7.78–10.45)	0.056	7.72 (6.96–8.93)	9.28 (8.26–10.26)	8.09 (6.82–9.39)	0.017
16	7.81 (6.63–8.52)	8.91 (8.01–10.36)	8.31 (7.52–10.12)	0.064	7.46 (6.59–8.39)	8.81 (7.78–9.86)	7.7 (6.45–9.05)	0.02
17	7.55 (6.57–8.23)	8.635 (7.54–10.08)	7.94 (7.19–9.77)	0.084	7.31 (6.31–7.83)	8.47 (7.27–9.55)	7.32 (6.14–8.79)	0.025
18	7.1 (6.49–8.23)	8.45 (7.36–9.77)	7.77 (6.90–9.4)	0.097	6.92 (6.16–7.31)	8.035 (6.88–9.12)	6.94 (5.85–8.61)	0.038
19	6.94 (6.31–8.22)	8.1 (7.20–9.55)	7.34 (6.57–8.94)	0.1	6.55 (6.02–6.86)	7.505 (6.4–8.73)	6.68 (5.4–8.18)	0.044
20	6.55 (5.93–7.98)	7.76 (6.64–8.99)	6.87 (6.04–8.34)	0.075	5.86 (5.65–6.29)	6.905 (6.09–7.95)	6.37 (4.99–7.52)	0.048
21	5.96 (5.27–7.3)	6.875 (5.88–8.02)	6.12 (5.25–7.39)	0.069	5.18 (4.82–5.5)	5.965 (5.41–6.97)	5.57 (4.46–6.61)	0.045
22	4.89 (4.28–6.04)	5.58 (4.87–6.55)	4.97 (4.19–6.03)	0.057	4.19 (3.81–4.45)	4.785 (4.27–5.51)	4.47 (3.60–5.26)	0.051
23	3.46 (3.01–4.3)	3.945 (3.44–4.59)	3.48 (2.92–4.27)	0.049	2.94 (2.63–3.12)	3.37 (2.93–3.88)	3.12 (2.52–3.67)	0.061
24	1.79 (1.54–2.23)	2.04 (1.78–2.36)	1.78 (1.49–2.21)	0.048	1.51 (1.34–1.61)	1.74 (1.50–2.01)	1.6 (1.29–1.90)	0.069

LV, left ventricle; RV, right ventricle; ED, end-diastole

^#^*p* < 0.05 GDM vs. DM

**Table 5:** Comparison of the right ventricular 24-segment FS Values among groups

RV 24-Segment FS Values (%)				
Variable	DM (*N* = 11)	GDM (*N* = 48)	Control (*N* = 25)	P
Segment 1	14.90 ± 8.69	10.73 ± 6.74^*^	16.71 ± 6.03	**0.002**
Segment 2	15.02 ± 8.34	10.88 ± 6.34^*^	16.38 ± 5.57	**0.002**
Segment 3	15.49 ± 7.45	11.16 ± 5.98^*^	16.13 ± 5.43	**0.002**
Segment 4	16.04 ± 6.61	11.47 ± 5.78^*^	15.92 ± 5.68	**0.004**
Segment 5	16.68 ± 5.87	11.93 ± 5.48^*#^	16.10 ± 5.15	**0.002**
Segment 6	17.38 ± 5.31	12.54 ± 5.09^*#^	16.35 ± 4.64	**0.001**
Segment 7	18.05 ± 5.07	13.10 ± 5.00^*#^	16.52 ± 4.54	**0.002**
Segment 8	18.58 ± 5.16	13.57 ± 5.19^*#^	16.56 ± 4.79	**0.004**
Segment 9	18.89 ± 5.47	13.90 ± 5.39^#^	16.44 ± 5.23	**0.012**
Segment 10	18.9 ± 5.93	13.97 ± 5.60^#^	16.14 ± 5.69	**0.026**
Segment 11	18.63 ± 6.56	13.87 ± 5.81	15.77 ± 6.11	0.052
Segment 12	18.12 ± 7.31	13.61 ± 6.11	15.39 ± 6.69	0.1
Segment 13	17.43 ± 8.08	13.30 ± 6.41	15.12 ± 7.48	0.176
Segment 14	16.65 ± 8.68	13.01 ± 6.56	15.08 ± 8.35	0.256
Segment 15	16.16 ± 8.62	12.76 ± 6.70	15.46 ± 8.83	0.223
Segment 16	16.03 ± 8.07	12.53 ± 7.01	15.94 ± 9.18	0.145
Segment 17	15.93 ± 7.98	12.43 ± 7.34	16.27 ± 9.38	0.116
Segment 18	16.13 ± 7.90	12.62 ± 7.42	16.23 ± 9.44	0.142
Segment 19	16.57 ± 7.83	12.90 ± 7.58	15.97 ± 9.26	0.196
Segment 20	16.96 ± 8.27	13.15 ± 7.99	15.78 ± 8.68	0.245
Segment 21	17.48 ± 8.45	13.36 ± 8.52	15.51 ± 8.31	0.277
Segment 22	17.99 ± 8.36	13.57 ± 8.84	15.28 ± 8.18	0.284
Segment 23	18.31 ± 8.35	13.70 ± 9.10	15.12 ± 8.16	0.283
Segment 24	18.49 ± 8.36	13.76 ± 9.25	15.02 ± 8.18	0.28

RV: right ventricle; FS: fractional shortening

^*^p < 0.05 GDM vs. control; ^#^*p* < 0.05 GDM vs. DM

**Table 6:** Comparison of the right ventricular 24-segment FS Z-scores among groups

RV 24-Segment FS Z-Scores				
Variable	DM (*N* = 11)	GDM (*N* = 48)	Control (*N* = 25)	P
Segment 1	0.51(0.18–1.77)	1.07(0.52–1.73)^*^	0.53(0.16–0.95)	**0.01**
Segment 2	0.7(0.09–1.7)	1.165(0.58–1.76)^*^	0.49(0.23–0.85)	**0.006**
Segment 3	0.69(0.04–1.6)	1.14(0.63–1.77)^*^	0.41(0.24–0.81)	**0.003**
Segment 4	0.63(0.08–1.48)	1.14(0.74–1.67)^*^	0.43(0.24–0.84)	**0.002**
Segment 5	0.65(0.11–1.39)	1.13(0.79–1.61)^*^	0.41(0.24–0.91)	**0.002**
Segment 6	0.66(0.18–1.22)	1.245(0.89–1.81)^*^	0.55(0.30–1.14)	**0.002**
Segment 7	0.61(0.36–1.04)	1.39(0.93–1.91)^*#^	0.74(0.53,1.38)	**0.001**
Segment 8	0.74(0.51–1.09)	1.57(0.96–2.05)^*#^	1.01(0.78–1.58)	**0.001**
Segment 9	0.73(0.46–1.21)	1.565(1.18–2.08)^#^	1.33(0.87–1.65)	**0.002**
Segment 10	0.85(0.49,1.34)	1.59(1.15–2.27)^#^	1.5(0.93–1.81)	**0.009**
Segment 11	0.92(0.37–1.52)	1.63(1.21–2.35)^#^	1.54(0.96–1.89)	**0.043**
Segment 12	0.9(0.39–1.64)	1.645(1.21–2.38)	1.49(0.96–1.90)	0.103
Segment 13	0.82(0.34–1.78)	1.615(1.15–2.34)	1.39(0.84–2.06)	0.207
Segment 14	0.76(0.28–1.93)	1.53(1.09–2.28)	1.31(0.70–2.11)	0.199
Segment 15	0.78(0.37–2.02)	1.395(1.11–2.19)	1.13(0.67–1.90)	0.165
Segment 16	0.77(0.32–1.72)	1.375(0.97–2.06)	1.04(0.59–1.74)	0.186
Segment 17	0.74(0.25–1.6)	1.445(0.78,2.06)	0.96(0.55,1.72)	0.209
Segment 18	0.76(0.27–1.43)	1.4(0.66–1.88)	0.96(0.44–1.65)	0.193
Segment 19	0.98(0.35–1.33)	1.43(0.81–1.99)	0.99(0.46–1.83)	0.175
Segment 20	0.99(0.42–1.06)	1.33(0.75–1.82)	0.88(0.44–1.63)	0.223
Segment 21	0.88(0.43–1.18)	1.28(0.66–1.73)	0.83(0.48–1.56)	0.278
Segment 22	0.76(0.39–1.21)	1.195(0.61–1.73)	0.82(0.64–1.47)	0.273
Segment 23	0.69(0.4–1.24)	1.15(0.58–1.72)	0.82(0.67–1.46)	0.279
Segment 24	0.65(0.38–1.25)	1.125(0.58–1.71)	0.81(0.67–1.44)	0.309

RV, right ventricle; FS: fractional shortening

^*^p < 0.05 GDM vs. control; ^#^*p* < 0.05 GDM vs. DM

### Relationships between maternal metabolic markers and fetal cardiac indices

Univariate analyses were performed on all the fetal echocardiography results of 11 DM and 48 GDM (*n* = 59) to explore the association of maternal characteristics and metabolic parameters with fetal cardiac indices.

The results showed a positive correlation between LV GLS with maternal age, a negative correlation between LV GLS with maternal pregestational weight and BMI, a negative correlation between maternal early pregnancy fast glucose with LV SV, LV CO, RV ED Area, RV ED Length, RV ES Area, RV ES Length, 4CV GSI, 4CV GSI%, 4CV ED, 4CV Length ED, and 4CV Area, a negative correlation between HbA1c with 4CV Width ED, 4CV Length ED, 4CV Area, and a negative correlation between total cholesterol and low-density lipoprotein cholesterol (LDL-Cholesterol) with RV GLS and RV FAC (Table 7).

**Table 7:** Correlations between maternal characteristics metabolism markers and fetal cardiac indices for DM and GDM patients

Variable	early pregnancy fast glucose (*N* = 59)	
	R	P value
LV SV(ml)	-0.317	**0.015**
LV CO(ml/min)	-0.321	**0.013**
RV ED Area (cm^2^)	-0.271	**0.038**
RV ED Length (cm)	-0.307	**0.018**
RV ES Area (cm^2^)	-0.309	**0.017**
RV ES Length (cm)	-0.338	**0.009**
4CV GSI	-0.277	**0.033**
4CV GSI% (%)	-0.277	**0.033**
4CV Width ED (mm)	-0.258	**0.048**
4CV Length ED (mm)	-0.379	**0.003**
4CV Area (mm^2^)	-0.350	**0.007**
	HbA1c	
	R	P value
4CV Width ED (cm)	-0.288	**0.029**
4CV Length ED (cm)]	-0.288	**0.028**
4CV Area (mm^2^)	-0.309	**0.018**
	total Cholesterol	
	R	P value
RV Global Strain(%)	-0.451	**0.000**
RV FAC(%)	-0.382	**0.003**
	LDL-Cholesterol	
	R	P value
RV Global Strain(%)	-0.376	**0.003**
RV FAC(%)	-0.311	**0.017**
	Age	
	R	P value
LV Global Strain(%)	0.029	**0.022**
	maternal pre-gestational weight	
	R	P value
LV Global Strain(%)	-0.307	**0.018**
	pre-gestational BMI	
	R	P value
LV Global Strain(%)	-0.264	**0.044**

N: the number of fetal echocardiography results of 11 DM and 48 GDM mothers and HbA1c data was not available for one GDM woman

LV, left ventricle; RV, right ventricle; GSI, global spherical index; ED, end-diastole; ES, end-systole; 4CV, 4 chamber view. FAC, fractional area change; EF, ejection fraction; SV, stroke volume; CO, cardiac output; HbA1c: hemoglobin A1c. LDL-Cholesterol: low-density lipoprotein cholesterol. BMI, Body mass index

R: Spearman correlation coefficient *P* < 0.05 was considered significant

## Discussion

Intrauterine hyperglycemia is an important environmental factor, which can affect the fetal heart in many different ways, including causing cardiac malformation in the early stages of pregnancy, interfering with metabolic and circulatory stability, and then causing myocardial remodeling in the middle and late stages of pregnancy, leading to hypertrophic cardiomyopathy and cardiac dysfunction.

Evaluation of fetal heart can assist identify alterations in heart structure, morphology, and function, enabling prompt monitoring and management in fetuses of hyperglycemia mothers [21, 22]. However, according to earlier research, the cardiac alterations are modest in hyperglycemic mothers’ fetuses, and might not be detectable using conventional echocardiographic methods [6, 23, 24].

In this study, we examined the morphometry and function changes in fetuses of women with pregestational and gestational diabetes mellitus using a novel technique of speckle tracking echocardiography—Fetal HQ, to quantitatively and thoroughly assess the fetal cardiac changes related to maternal complications. We discovered that fetuses of GDM mothers have compromised cardiac geometry and function, which may be a response to an intrauterine environment with relative hyperglycemia during diabetes pregnancy.

In our study, the cardiac morphological parameters change, particularly in the left ventricle, in fetuses from the GDM group, demonstrating an increase in ventricular size and volume, representing fetal heart dilation. While in the DM group, the alteration was insignificant in comparison to the controls, which may be due to the different pathophysiology between DM and GDM.

GSI, as the ratio of the total longitudinal length to the transverse length of the 4CV at end-diastole, has been suggested as a screening tool for assessing cardiac shape since it allows for the evaluation of the overall deformation and movement of ventricles [13]. In our study, the lower GSI in the fetus of diabetic groups signified the remodeling of the heart from elliptical to spheroidal, suggesting that the whole heart was dilated as a result of the hyperglycemia surroundings. Our findings are in line with some earlier studies that diabetic fetuses had rounder hearts [25–27].

The size and shape of the heart were directly related to its function. We discovered that the LV SV and CO of fetuses in the GDM group were significantly higher compared to the DM group, and insignificantly higher than those in the control group, whereas the alteration of them was in the opposite trend in the DM groups, but insignificant as compared to the controls, indicating the different influence of hyperglycemia on fetal cardiac contractility between DM and GDM.

As the fetal heart requires glucose for energy, when ischemia or hypoxia occurs, the endocardium is the first to be injured coupled with a decrease in GLS, making it a sensible marker for early identification of cardiac systolic function impairment [13]. Previous research has shown that gestational and/or pregestational diabetes reduces RV GLS in fetuses [6, 23, 28], although there is conflicting evidence regarding LV GLS. Song et al. [27] studied 60 fetuses with maternal diabetes (MD) and 60 controls between 19 and 37 gestational weeks and revealed that the RV GLS was considerably lower in the DM group. Yovera et al. [25] examined 112 fetuses of women with GDM at 24 to 32 weeks and 32 + 1 to 40 + 1 weeks and reported a significant reduction of RV GLS but normal LV GLS in both periods. Besides, only a decrease in RV GLS was discovered when Miranda et al. [6] evaluated 76 fetuses of women with GDM at 30 to 33 gestational weeks. In contrast, Kulkarni et al. [29] demonstrated a decrease in LV GLS in fetuses of 51 pregestational diabetes and 31 GDM women at 20–30 weeks’ gestation; Patey et al. [28] also reported comparable results. As a new technique in China, there was only a few domestic studies concerning fetal HQ application in GDM, Li et al. [30] compared myocardial function of fetuses from 47 GDM women and 62 healthy controls at 24 to 28 weeks found that the LV GLS of GDM was lower, and the LV GLS independently associated with GDM, thus they pointed out LV GLS may serve as an indicator of subclinical systolic dysfunction of GDM. Huang et al. [3]studied cardiac systolic function of 49 fetuses exposed to GDM and 50 normal fetuses, also discovered that the LV and RV GLS of the GDM group were significantly lower compared with the control group. Our findings indicated that fetuses of diabetic mothers tended to have lower RV GLS and higher LV GLS, which reflects reduced right ventricular cardiac performance, but the differences were not statistically significant. These discrepancies between our study and others may be partly explained by the appropriate pre-pregnancy BMI and satisfactory glycemic control of our subjects and may also be attributed to the inclusion of different types of diabetes, variation in maternal characteristics, gestational age at ultrasound examination, management of diabetes, image quality, and various speckle-tracking software, which highlights the demand for more research.

FAC is regarded as an appropriate indicator when assessing ventricular contractility as it corresponds well with both longitudinal and transverse contraction [13]. According to Yovera’s study [25], the RV FAC of fetuses of GDM mothers was much lower. Wang et al [26] investigated fetuses of 58 GDM women and discovered that FAC in LV and RV considerably decreased at both 24–27 + 6 weeks and 28–40 weeks. Song et al. [27] also discovered that the RV FAC was significantly lower in fetuses of diabetes mothers between 19 and 37 gestational weeks. In our study, although the RV FAC tended to decline in fetuses of diabetic mothers, there were no statistically significant differences between the three groups. Further large-sample studies are required to evaluate the value of the slight differences observed.

We then explored the correlations between maternal characteristics, glucose, and lipid metabolism with fetal cardiac changes, and our findings indicated that maternal pregestational weight and BMI, early pregnancy fast glucose, lipids, and glycemic control levels influence fetal cardiac morphology and function. The relatively hyperlipidemic intrauterine environment may overstimulate fetal pancreatic beta cells, resulting in fetal hyperinsulinemia and inducing oxidative stress and inflammatory reactions. These events may then increase proinflammatory mediators of the myocardium, promote myocardial connective tissue accumulation, and affect myocardial fiber architecture [7, 31], additionally, fetal hyperinsulinemia can increase metabolic rate and have an increased tendency toward volume overload and hypoxia [32], contractility compensatory increase in the early stages, then after a limited time, causing cardiomyocyte injury, accumulation of collagen and local fibrosis, and even apoptosis [7, 8, 32, 33], resulting in cardiac deformation and dysfunction [12], which may explain the increase in volume and cardiac contractility of GDM fetuses, while the opposite alteration trend in fetuses of DM, which suffered from the unfavorable metabolic environment for a longer time. Besides, according to our findings, maternal metabolic control may be helpful in risk modification of the impairment of the fetal heart, and further research is demanded in this field.

Fetal HQ can perform a 24-segment myocardial analysis to assess the small morphological and functional changes in each segment of the heart and is therefore more sensitive and comprehensive. Both Huang et al [3]. and Chen et al [5]. found that the 24-segment transverse FS of the RV reduced in the GDM group. In accordance with recent cardiac deformation studies that used a similar methodology [3, 5], our study demonstrated impaired transverse contractility for RV segments 1–7 in fetuses exposed to GDM, as reflected by an FS value significantly lower than the control, which demonstrates RV systolic dysfunction in GDM-exposed fetuses.

Our findings indicate that the fetal right ventricle is more likely than the left to have compromised cardiac function. Previous research has demonstrated that the RV plays a major role in fetal circulation and that its systolic function is more vulnerable to damage than the LV [34]. Since the fetal heart depends on glucose metabolism, hyperglycemia may cause the fetus’s metabolism to accelerate, and induced hypoxia and increased oxidative stress would initially harm the RV, which may account for decreased cardiac performance [4].

The exact mechanism by which maternal hyperglycemia affects fetal cardiac morphology and function is still unknown, prior research has shown that the various mechanisms share many pathogenic pathways, including metabolic disturbance and hyperinsulinemia, dysregulation of the insulin-like growth factors [32, 35], oxidative stress [36], and inflammatory pathways [32], changes in loading conditions and fetal hypoxemia, which cause cardiomyocyte injury and apoptosis, resulting in myocardial deformation and dysfunction [7, 8, 12, 32, 33, 37], which are yet to be defined completely. While in the clinical domain, advanced imaging allows us to show the morphological and functional characteristics of the fetal heart. Fetal HQ, as a new two-dimensional speckle tracking technology, can track fetal myocardial speckles over the course of the heartbeat cycle with less angle dependence and the ability to objectively quantify myocardial deformation, which can be used to simultaneously estimate the shape, size, and contractility of the biventricular chambers [38–41]. It provides improved detection of mild cardiac dysfunctions, is a promising tool for examining early subclinical heart changes occurring in utero, offers insights into the effects of maternal diseases, and may also have good potential in developing into a surveillance method for detecting fetal deterioration. In our study, biventricular global deformation and changes in cardiac function were comprehensively described in the fetuses of diabetic mothers using fetal HQ. Lower GSI suggests that the entire fetal heart is dilated, and lower RV GLS and FAC suggest the impairment of the RV systolic function, indicating the effects of maternal hyperglycemia.

Although the application of fetal HQ evaluating fetal cardiac deformity and function in maternal and fetal diseases has been more common worldwide, it is still a new technique in China, and only a few publications concerning its application in maternal diabetes, which showed similar results to ours. Few studies like ours have associated maternal characteristics and metabolism of glucose and lipid with fetal cardiac changes, despite Li et al. [30] included clinical parameters such as maternal BMI and blood glucose in their study, the effects of these factors on fetal cardiac function and the relationship with the echocardiographic characteristics presented by the new technique was not explored. According to our results, optimal maternal metabolic control, such as lower early pregnancy glucose and lipids, and normal preconception body weight control is beneficial as these may be associated with fetal cardiac function. Besides, our findings imply that fetal HQ is a promising technique for identifying cardiac malformations and subclinical systolic myocardial dysfunction in fetuses of GDM mothers. Early and subtle changes identified may be beneficial in anticipating unfavorable outcomes, and using a fetal period as a potential window for interventions, optimizing the perinatal outcomes of diabetes fetuses.

The limitation of this study is its small sample size for both the study and control groups, which could result in statistically insignificant findings in the DM group. The result of the study suggests further larger prospective trials employing fetal HQ to evaluate fetal morphology and function of hyperglycemia mothers. Besides, the majority of the pregnant women with DM and GDM in our study were with suitable pre-pregnancy BMI and adequate glycemic control, making it impossible to assess the influences of maternal high glycemic levels and obesity or with other inflammatory conditions on the shape and function of the fetal myocardium and the application value of Fetal HQ. Furthermore, we did not follow up with the fetuses post-natally after birth. A further extensive study of fetal cardiac morphology and function changes caused by different maternal glycemic control level and BMI, including long-term follow-up is required to supplement the effects of glycemia on fetal cardiac changes accessing by fetal HQ.

## Conclusions

Fetuses exposed to gestational diabetes exhibit impaired cardiac morphology and function and are associated with maternal age, pregestational weight, and metabolism of glucose and lipids, which represents a special indication for performing geometry and function cardiac assessment.

Fetal HQ can be used to identify sub-clinical cardiac morphology, size, and function changes in the fetus of diabetic mothers. A further larger prospective trial with long-term follow-up should be carried out using fetal HQ, to clarify the adverse effects of maternal hyperglycemia on fetal cardiac morphology and function during pregnancy as well as in postnatal life, and evaluate the impact of maternal glucose intervention on the fetal myocardial changes and the outcome of the fetuses in the short-term as well as in the long run, so that through refining pregnancy management and therapeutic strategies, optimizing perinatal and long-term outcomes of diabetic pregnancy.

## Data Availability

All data generated or analyzed during this study are included in this article. Further enquiries can be directed to the corresponding authors.

## Abbreviations

DM: diabetes mellitus
GDM: gestational diabetes mellitus
STE: speckle-tracking echocardiography
GSI: global sphericity index
GLS: global longitudinal strain
FAC: fractional area change
LV: left ventricle
RV: right ventricle
4CV: 4 chamber view
EF: ejection fraction
SV: stroke volume
CO: cardiac output
EDD: end-diastolic diameter
ED: end-diastole
ES: end-systole
FS: fractional shortening
SI: sphericity index
HbA1c: hemoglobin Alc
BMI: body mass index
HDL-Cholesterol: high-density lipoprotein cholesterol
LDL-Cholesterol: low-density lipoprotein cholesterol
